# A Compensatory-Topology and Relay-Gating Graph Network for multimodal stroke rehabilitation assessment

**DOI:** 10.3389/fphys.2026.1867188

**Published:** 2026-07-30

**Authors:** Haoge Zhu, Boyuan Wang

**Affiliations:** 1 School of Information Engineering, Capital Normal University, Beijing, China; 2 Center for Medical AI Technology Innovation, Zhuhai Fudan Innovation Research Institute, Zhuhai, China

**Keywords:** action-quality assessment, compensatory movement, inertial measurement unit, multimodal fusion, skeleton sequence, ST-GCN, stroke rehabilitation assessment

## Abstract

Accurate assessment of motor function is central to stroke rehabilitation. However, automated assessment remains challenging because task completion must be distinguished from compensatory movement, and skeletal and inertial sensors capture different aspects of movement. We propose CTCG-Net, a multimodal spatio-temporal graph framework for therapist-assisted rehabilitation assessment. The framework combines a compensation-aware Prior-Guided ST-GCN with Kinematic Relay Gating, which uses synchronised IMU dynamics to modulate skeletal features. A Spatially Decoupled Regression Head estimates Primary Outcome (PO) and Control Factor (CF) scores separately. On a public multimodal rehabilitation benchmark, CTCG-Net achieved the lowest average errors among the evaluated methods, with a MAD of 0.4001 and an RMSE of 0.5026. Ablation, agreement, robustness, topology, and saliency analyses indicated that the compensation-aware topology and relay gating contributed complementary information to PO and CF estimation. CTCG-Net is intended to support quantitative, clinician-supervised rehabilitation monitoring rather than autonomous diagnosis.

## 1 Introduction

Stroke is a major cause of long-term disability and often results in persistent motor impairment that affects activities of daily living and quality of life (Santisteban et al., 2016; Baird et al., 2025). Rehabilitation planning therefore requires repeated assessment of movement quality. Such assessment must consider both task completion and the manner in which the movement is performed (Liao et al., 2020b; Rahman et al., 2023; Khalaf et al., 2026). Therapist-administered scales and structured observations remain clinically indispensable, but they are time-consuming, partly subjective, and difficult to standardise for frequent follow-up or home-based monitoring. These limitations motivate computational methods that provide reproducible quantitative evidence while preserving clinical interpretation and decision-making.

Sensor-assisted methods have broadened the scope of rehabilitation assessment. Depth cameras and vision-based motion-capture systems characterise whole-body posture and joint trajectories. Wearable IMUs record local acceleration and angular velocity, whereas physiological sensors provide complementary information on muscle activation and neural control (Liao et al., 2020b; Rahman et al., 2023; Khalaf et al., 2026; Islam, 2024; Ma et al., 2025). However, additional sensing alone does not resolve the assessment problem. Skeletal streams encode anatomical configurations, whereas IMU streams emphasise local dynamics. Premature fusion can obscure the contribution of each modality to a clinical score.

The central modelling problem therefore has two components. First, the model must represent long-range compensatory coordination that a purely anatomical skeletal graph may not capture. Second, it must preserve the distinction between task-oriented movement accomplishment and compensation-related control burden. In this study, Primary Outcome (PO) and Control Factor (CF) scores represent these dimensions. Separate estimation of PO and CF better reflects the clinical distinction between successful task execution and the movement strategy used to achieve it.

**Figure 1:**
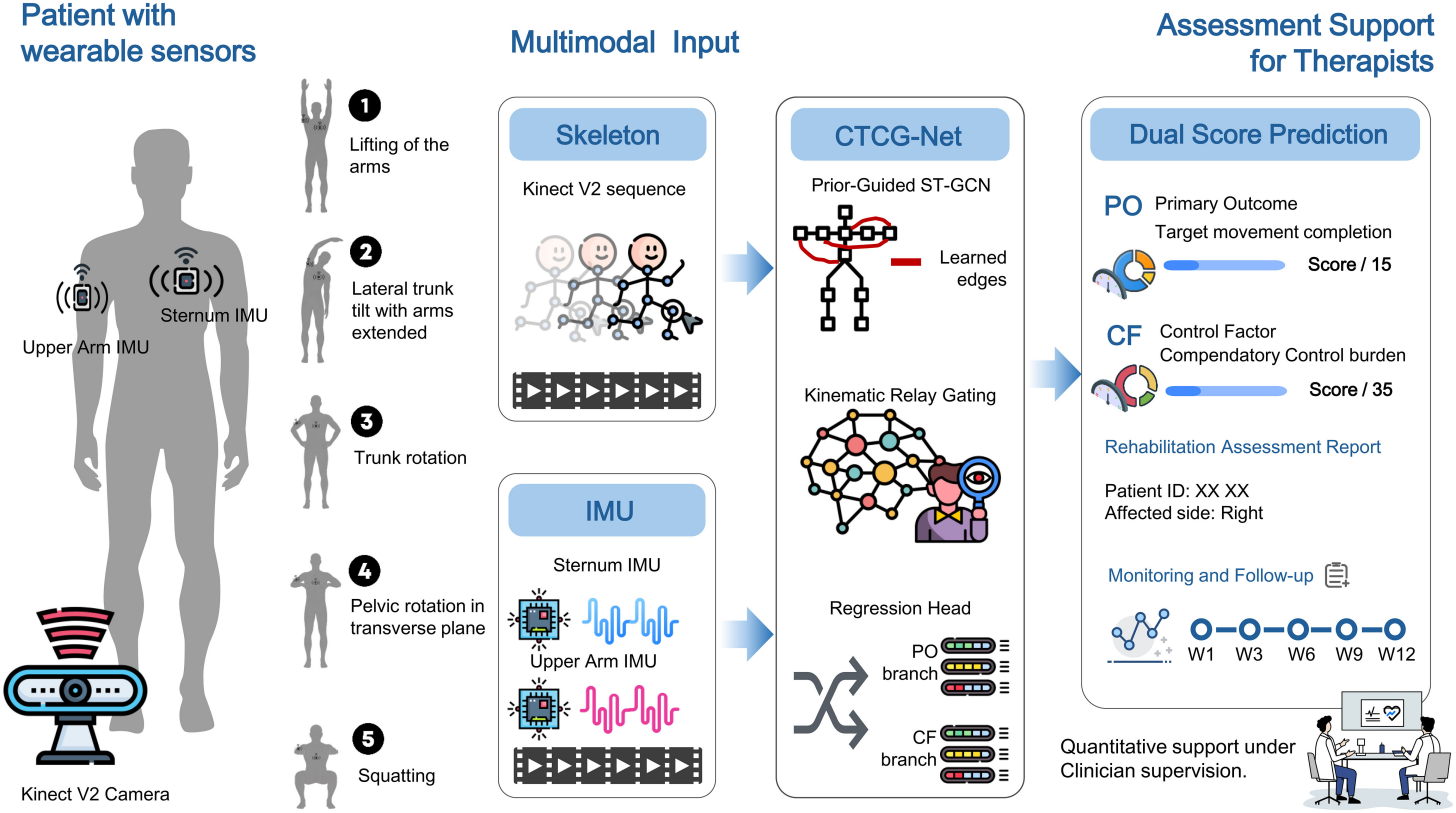
Overall study workflow. The pipeline comprises movement acquisition using Kinect-based skeletal tracking and wearable IMUs, multimodal feature processing by CTCG-Net, and generation of PO and CF scores for therapist-assisted interpretation.

To address these challenges, we developed CTCG-Net, a multimodal spatio-temporal graph framework for stroke rehabilitation assessment. As shown in **Figure 1**, the workflow links sensor-based movement acquisition, multimodal feature modelling, and therapist-oriented score estimation. The framework is designed for use under clinician supervision. It provides standardised quantitative references for movement analysis and longitudinal follow-up without replacing professional clinical judgement.

The contributions of this study are threefold.

First, CTCG-Net augments the anatomical skeletal graph with a learnable, compensation-aware topology that explicitly represents long-range, cross-regional coordination.

Second, a lightweight relay-gating mechanism incorporates time-varying IMU dynamics while preserving the structural interpretability of skeletal features.

Third, a spatially decoupled regression head estimates PO and CF as related but distinct rehabilitation targets. We evaluated this design through baseline comparisons, ablation experiments, loss-weight sensitivity analysis, corrupted-input robustness testing, topology visualisation, and branch-specific saliency analysis.

## 2 Related work

Recent studies increasingly frame stroke monitoring as a technology-assisted, data-driven process rather than an episodic clinical scoring task. Reviews of automated rehabilitation assessment, depth-camera analysis, telerehabilitation, robot-assisted therapy, and physiological-signal platforms highlight the potential of quantitative monitoring. They also emphasise the need for standardised protocols, clinically meaningful outputs, and human oversight (Rahman et al., 2023; Zhou et al., 2025c; Khalaf et al., 2026). Clinical and informatics studies similarly suggest that remote outcome assessment and causal machine learning can support rehabilitation monitoring when outputs remain interpretable and aligned with clinician feedback (Jordan et al., 2024; Baird et al., 2025). The unresolved question therefore extends beyond prediction accuracy to whether the computational representation reflects the clinical structure of movement-quality assessment.

Wearable and multimodal sensing methods provide complementary evidence for this purpose. Accelerometry and IMU-based systems have quantified upper-extremity use, home-practice quality, upper-limb kinematics, gait, and lower-limb movement after stroke (Geed et al., 2023; Seo et al., 2024; Cinnera et al., 2024; Bishop et al., 2024; Lee et al., 2024; Guo et al., 2025; Zhou et al., 2025b). More recent learning-based studies have estimated Fugl-Meyer Assessment scores or impairment-related movement quality from wearable or multimodal sensor streams (Di Pietro et al., 2025; Liu et al., 2025; Zhou et al., 2025a). These studies indicate that sparse sensors can capture clinically relevant motion information. However, many models focus on activity quantity, global impairment scores, or single-modality prediction. Therapist-oriented assessment therefore requires a method that combines local inertial dynamics with global skeletal configurations while retaining an interpretable representation of body-segment coordination.

Skeletal action-quality assessment has advanced rapidly in physical rehabilitation because skeletal sequences provide compact, anatomically structured representations of human movement. Public datasets such as KIMORE and UI-PRMD established early benchmarks for rehabilitation movement scoring (Capecci et al., 2019; Vakanski et al., 2018; Islam, 2024). Recent methods have improved exercise assessment through contrastive representation learning, dense spatio-temporal graph modelling, lightweight temporal convolution, ensemble graph fusion, and multiview skeletal representations (Yao et al., 2023; Mourchid and Slama, 2023; Bai et al., 2023; Sardari et al., 2024; Yu et al., 2024; Kuang et al., 2025). Other studies have examined upper-limb rehabilitation using posture and force information, frame-topology fusion, and home-oriented ST-GCN attention models (Bai et al., 2024; Zhang et al., 2025; Lim et al., 2026). Despite these advances, most methods treat rehabilitation quality as a single score or an exercise correctness regression problem. This formulation may obscure the distinction between task achievement and compensatory movement strategies.

Graph-based skeletal modelling provides the technical foundation for many of these approaches. ST-GCN and subsequent skeletal action-recognition models have shown that graph convolution represents joint relationships and temporal dynamics more naturally than conventional vector representations of joint coordinates (Yan et al., 2018; Chen et al., 2021; Duan et al., 2022; Qian, 2024). However, rehabilitation assessment differs from generic action recognition. Clinically relevant errors may involve long-range compensatory coupling, such as trunk recruitment during impaired upper-limb movement, rather than only local anatomical dependencies. Existing graph-based rehabilitation models improve feature extraction and scoring accuracy, but compensation-related topology and controlled IMU-to-skeleton modulation remain underdeveloped. CTCG-Net addresses this gap through a compensation-aware graph prior, IMU-guided relay gating, and a spatially decoupled regression head for separate PO and CF estimation.

## 3 Materials and methods

### 3.1 Study design and intended clinical use

This retrospective methodological study evaluated a multimodal learning framework for therapist-assisted stroke rehabilitation assessment. We tested whether synchronised skeletal and IMU streams could estimate two related movement-quality targets, PO and CF, under a participant-level evaluation protocol. The intended application is clinician-supervised monitoring. Model outputs serve as auxiliary quantitative references for movement analysis and follow-up comparison, not as independent bases for diagnosis or treatment decisions. The final implementation details and configuration of CTCG-Net are summarized in **Table 1**.

**Table 1:** Implementation details and final configuration of CTCG-Net.

Item	Final configuration
Optimizer and schedule	AdamW; initial learning rate 5×10^−4^; weight decay 1×10^−4^; step decay at epochs 30, 50, and 70 with *γ* = 0.1
Batch size and epochs	Mini-batch size 16; 100 training epochs; evaluation batch size 32
Data partition	Participant-level train/validation/test split of 101/13/12 participants using split seed 42; all trials from one participant were assigned to the same split
Input representation	Skeleton sequence length 512; 25 joints; 3 skeletal channels; 2 IMU sensors with 9 channels per sensor, yielding 18 synchronised inertial channels
Backbone depth and channels	Six CTCG blocks with channel widths [64, 64, 64, 128, 128, 256] and temporal kernel size 9
Hidden dimensions	IMU temporal encoder hidden dimension 64; PO and CF regression-head hidden dimension 64
Graph construction	Kinect/NTU 25-joint layout; spatial three-partition ST-GCN graph prior; learnable compensation residual topology with compensation scale 0.15
Regularisation and gating	Dropout 0.35; edge-importance weighting enabled; relay-gate scale 0.35; gradient clipping norm 5.0; mixed-precision training enabled
PO mask nodes	[5, 6, 7, 9, 10, 11, 13, 14, 15, 17, 18, 19, 21, 22, 23, 24]
CF mask nodes	[0, 1, 2, 3, 4, 8, 12, 16, 20]
Loss function and weights	Smooth L1 loss for PO and CF plus L1 sparsity on the compensation topology; *λ*_po_ =1.0, *λ*_cf_ =1.0, *λ_s_* = 1×10^−4^
Hyperparameter selection	Candidate settings were selected using the validation set under the fixed participant-level split; the held-out test set was used only for final reporting
Model size and computation	2,367,676 trainable parameters (2.3677M); 12.1561 GFLOPs per 512-frame trial, equivalent to 23.7425 MFLOPs per frame

FLOPs were estimated for one 512-frame multimodal trial. Node indices follow the zero-based Kinect/NTU skeletal layout used in the implementation.

### 3.2 Dataset, participants, and ethics

We used a public stroke rehabilitation exercise dataset containing Kinect-based skeletal sequences, IMU recordings, participant information, and clinician-assigned PO and CF scores (Islam, 2024). Related public datasets include KIMORE (Capecci et al., 2019) and UI-PRMD (Vakanski et al., 2018), which are widely used for movement-quality assessment and rehabilitation exercise analysis. The public release comprises 128 participants and five predefined rehabilitation exercises.

Available participant metadata were limited to participant identifier, age, and sex. The release did not include time since stroke, lesion side, rehabilitation stage, motor severity, or standardised neurological scale scores. We therefore treated the dataset as a public multimodal benchmark for rehabilitation movement assessment rather than a fully characterised clinical stroke cohort. This distinction limits the translational scope and generalisability of the model.

The public repository states that the institutional review board of Independent University, Bangladesh, approved data acquisition and scoring. However, the release does not provide a verifiable approval number, a detailed recruitment protocol, complete exclusion criteria, or a companion clinical methods paper describing the labelling procedure. The present study relies exclusively on the released benchmark files and does not claim access to unpublished clinical information.

### 3.3 Rehabilitation exercises and clinical relevance

Five standard rehabilitation exercises were analysed in this study:

1. Arm lifting, which primarily reflects upper-limb range of motion, bilateral coordination, movement symmetry, and compensatory trunk involvement.
2. Lateral trunk tilt with the arms extended, which challenges trunk control, upper-limb extension, lateral stability, and postural adjustment.
3. Trunk rotation, which reflects axial mobility, inter-segmental coordination, and dissociation between upper- and lower-body motion.
4. Pelvic rotation in the transverse plane, which emphasises pelvic control, core stability, and transverseplane coordination.
5. Squatting, which assesses lower-limb functional movement, postural control, trunk alignment, and global movement stability.

These categories constitute the complete set of predefined rehabilitation tasks in the public benchmark. Inclusion of all available exercises avoided selective task exclusion. It also enabled evaluation across upper-limb-dominant, trunk-oriented, pelvic-control, and lower-limb or postural-control movements within the same dataset.

### 3.4 Reference standard and label definition

We formulated rehabilitation assessment as a two-dimensional supervised regression task using two exercise-level scores provided with the dataset: Primary Outcome (PO) and Control Factor (CF). PO represents the extent to which the intended movement objective is achieved. CF reflects compensation-related control burden during movement execution, including abnormal trunk substitution, excessive proximal recruitment, and postural compensation.

PO and CF were therefore interpreted jointly in therapist-assisted assessment. A high PO score indicates successful task completion but does not necessarily indicate high-quality control when CF identifies substantial compensatory burden. The outputs provide complementary references: PO reflects movement accomplishment, whereas CF indicates whether compensatory strategies contributed to that accomplishment.

Because the target dataset does not provide a detailed scoring manual, we aligned the clinical interpretation of PO and CF with the framework introduced in KIMORE (Capecci et al., 2019). In KIMORE, clinicians evaluated rehabilitation exercises using the Exercise Accuracy Assessment Questionnaire (EAAQ). The first three items assess achievement of the primary motor goal and form the Primary Outcome score. The remaining items assess posture-related control across body segments and form the Control Factors score. We used KIMORE only to clarify the clinical meaning of PO and CF, not to imply direct equivalence between the datasets.

The public release does not disclose the number or professional background of the raters. It also does not report whether scoring was independent or blinded, how disagreements were resolved, or whether inter-rater reliability was established. We therefore treated PO and CF as benchmark supervisory signals rather than labels from a fully documented clinical reference-standard protocol. The model reproduces the released annotations under the benchmark conditions; these annotations should not be considered fully validated clinical ground truth. We accounted for this limitation when interpreting the results.

### 3.5 Data acquisition and preprocessing

The dataset contained two synchronised sensing modalities (Islam, 2024). The visual stream comprised three-dimensional skeletal motion captured with Kinect V2. The inertial stream comprised two IMUs placed on the lateral aspect of the paretic upper arm and on the sternum. Each IMU recorded triaxial acceleration, angular velocity, and magnetometer signals. We temporally aligned the multimodal sequences and converted them into fixed-length representations for batch training and graph-based modelling.

Preprocessing comprised motion-sequence cleaning, temporal normalisation, and frame-count standardisation. Because recording durations varied, we resampled all trials to 512 frames for temporal pooling and mini-batch optimisation. After preprocessing, the skeletal stream comprised temporal sequences of joint features, and the IMU stream comprised synchronised multichannel inertial measurements.

Fixed-length standardisation improved optimisation stability and ensured consistent input dimensions across models. However, this procedure is an offline modelling choice rather than a solution for real-time deployment. Variable-length and streaming inference would require additional development.

### 3.6 Proposed method: CTCG-Net

**Figure 2:**
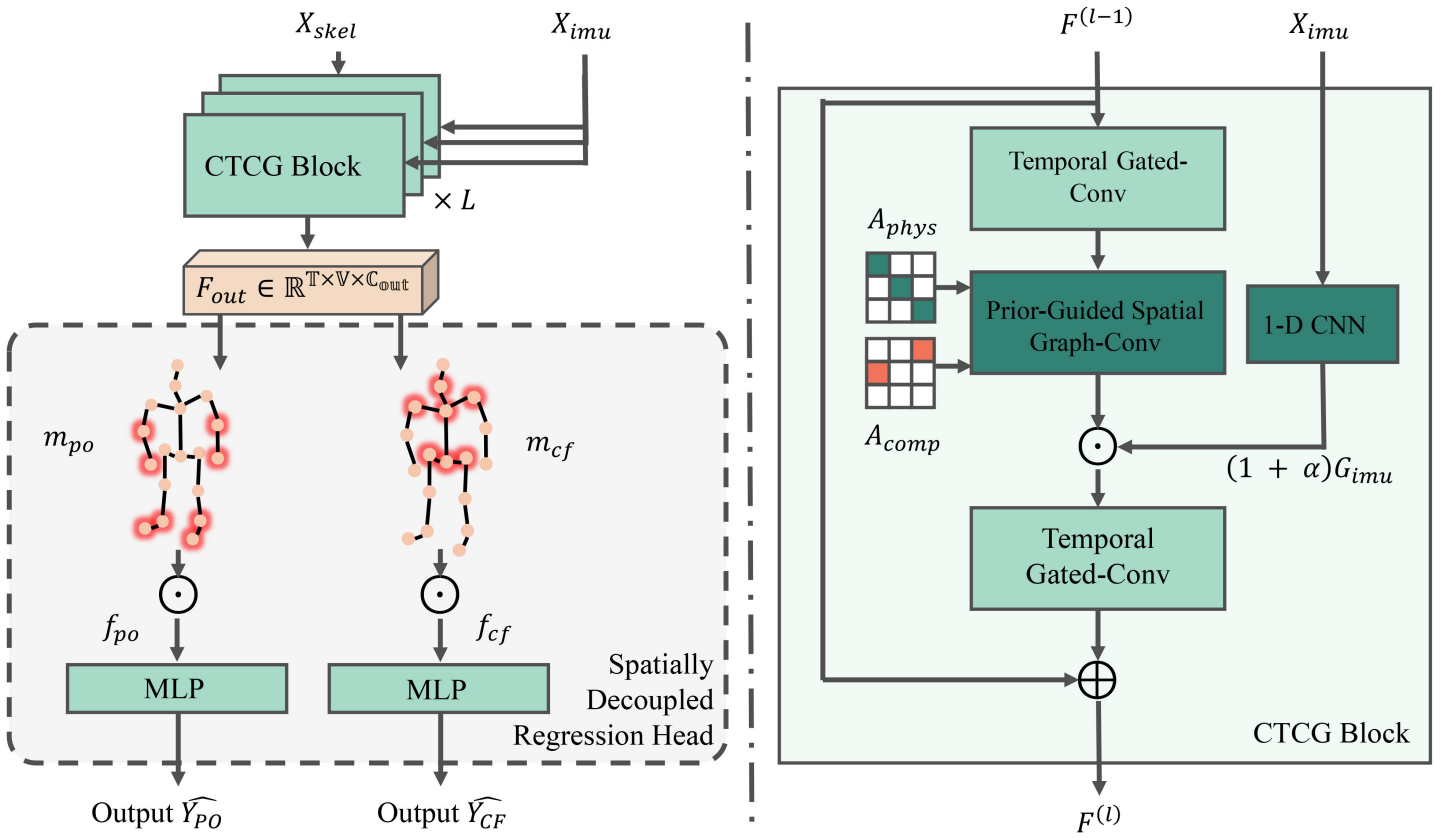
Architecture of CTCG-Net. The framework combines compensation-aware graph convolution of skeletal sequences, inertial-guided relay gating, and spatially decoupled regression branches for PO and CF estimation.

CTCG-Net processes synchronised skeletal and inertial sequences to estimate PO and CF scores for each exercise trial. As shown in **Figure 2**, the framework comprises a Prior-Guided ST-GCN backbone, a Kinematic Relay Gating module, and a Spatially Decoupled Regression Head. These components address compensation-aware graph reasoning, controlled multimodal interaction, and clinically meaningful separation of the assessment targets.

The inputs are a skeletal motion sequence *X_s_*and a synchronised inertial sequence *X_i_*. Here, *T* denotes the temporal length, *V* the number of skeletal joints, *C_s_*the skeletal feature dimension, and *C_i_*the inertial feature dimension. The model outputs continuous PO and CF scores for each movement trial.

Unlike action-quality models that regress a single overall score, CTCG-Net separates movement completion quality from compensation-related control burden. The Compensation-Aware Prior-Guided ST-GCN models topological movement dependencies. The Kinematic Relay Gating module uses IMU dynamics to regulate skeletal features, and the Spatially Decoupled Regression Head aggregates branch-specific spatial evidence for PO and CF prediction. The main computations of CTCG-Net are defined in Equations 1–6.

#### 3.6.1 Compensation-aware prior-guided ST-GCN

Conventional ST-GCNs model human motion by propagating information through local anatomical connections. Post-stroke rehabilitation movements may also involve long-range compensatory coupling, such as trunk recruitment during impaired upper-limb movement. To represent these dependencies, CTCGNet augments the anatomical skeletal graph with a learnable, compensation-aware topology.

Let $H^{\left(l\right)}\in {\mathbb{R}}^{T\times V\times C_{l}}$ denote the node features at the l-th graph convolution layer. The graph update is formulated as

(1) $$H^{\left(l+1\right)}=\sigma (\sum_{k=1}^{K}{\hat{A}}_{k}^{\left(l\right)}H^{\left(l\right)}W_{k}^{\left(l\right)}),$$

where $W_{k}^{\left(l\right)}$ is the learnable projection matrix for partition *k*, *σ*(·) is the nonlinear activation function, and ${\hat{A}}_{k}^{\left(l\right)}$ is the normalised adjacency matrix used for message propagation.

To incorporate clinically motivated compensation patterns, the effective adjacency is defined as

(2) $${\hat{A}}_{k}^{\left(l\right)}={\hat{A}}_{k}^{\text{phy}}+\text{Δ}A_{k}^{\text{comp},(l)},$$

where ${\hat{A}}_{k}^{\text{phy}}$ is the normalised anatomical adjacency prior and $\text{Δ}A_{k}^{\text{comp},(l)}$ is a learnable, compensation-aware residual topology. The backbone therefore preserves anatomical connectivity while capturing pathological cross-regional coordination that the original skeletal graph does not represent.

Abnormal rehabilitation movements can involve both local joint interactions and compensatory patterns across distant body regions. Embedding these dependencies in the graph structure encourages the model to learn rehabilitation-oriented movement representations rather than generic action descriptors.

#### 3.6.2 Kinematic relay gating

Skeletal motion provides a global description of body configuration, whereas inertial measurements may capture subtle dynamics associated with instability, effort, or compensation more directly (Kim et al., 2025; Hu et al., 2025; Song et al., 2026; Ma et al., 2025). Direct early fusion may entangle modality-specific features and reduce interpretability. CTCG-Net instead uses IMU features to generate a dynamic gating signal that modulates the graph-based skeletal representation.

Given the synchronised inertial sequence *X*_imu_, the temporal encoder Φ_imu_(·) generates a gating vector:

(3) $$G_{\text{imu}}=\sigma_{\text{sig}}({\text{Φ}}_{\text{imu}}(X_{\text{imu}})),$$

where $\sigma_{\text{sig}}(\cdot )$ is the sigmoid activation and $G_{\text{imu}}\in {\mathbb{R}}^{T\times d_{g}}$ is the time-varying inertial guidance signal.

The gating signal is then projected into the graph feature space and modulates the skeletal representation as follows:

(4) $${\tilde{H}}^{\left(l\right)}=H^{\left(l\right)}⊙(1+\text{Ψ}(G_{\text{imu}})),$$

where Ψ(·) is a learnable projection from the inertial gating space to the graph feature space, and ⊙ denotes element-wise multiplication. The inertial stream therefore acts as a kinematic regulator that dynamically modulates graph features without forcing both modalities into a fully shared latent space.

This relay-gating strategy allows local inertial evidence to inform skeletal movement modelling while preserving the structural interpretability of the graph backbone.

#### 3.6.3 Spatially decoupled regression head

In clinical rehabilitation assessment, a single scalar does not fully characterise movement quality. A patient may complete the target action while relying extensively on compensatory trunk motion or abnormal proximal stabilisation. To preserve this distinction, the final graph representation is decomposed into spatially biased subspaces for Primary Outcome (PO) and Control Factor (CF).

Let $F_{\text{out}}\in {\mathbb{R}}^{T\times V\times C}$ denote the output feature tensor of the final graph layer. Two clinically informed masks, *M*_po_ and *M*_cf_, emphasise different skeletal regions. The decoupled pooled features are computed as follows:

(5) $$f_{\text{po}}=\text{Pool}(M_{\text{po}}⊙F_{\text{out}}),\;\;f_{\text{cf}}=\text{Pool}(M_{\text{cf}}⊙F_{\text{out}}),$$

where Pool(·) denotes temporal-spatial pooling. The mask *M*_po_ emphasises effector and execution-related joints. The mask *M*_cf_ emphasises the trunk, pelvis, shoulder girdle, and other regions associated with compensation and postural control.

The two pooled representations are then passed to separate regression heads:

(6) $${\hat{y}}_{\text{po}}=\phi_{\text{po}}(f_{\text{po}}),\;\;{\hat{y}}_{\text{cf}}=\phi_{\text{cf}}(f_{\text{cf}}),$$

where ϕpo(·) and ϕcf(·) are lightweight, fully connected regressors for PO and CF, respectively.

Separating the final feature-aggregation process encourages the model to preserve complementary information on task accomplishment and compensation-related control burden instead of collapsing both targets into an undifferentiated latent representation.

#### 3.6.4 Objective function

The model was trained by jointly optimising the PO regression loss, the CF regression loss, and a sparsity penalty on the compensation-aware graph residual. The overall objective was defined as follows:

(7) $$L=\lambda_{\text{po}}L_{\text{po}}+\lambda_{\text{cf}}L_{\text{cf}}+\lambda_{s}∥\text{Δ}A^{\text{comp}}{∥}_{1},$$

where $L_{\text{po}}$ and $L_{\text{cf}}$ are the regression losses for PO and CF, respectively. The term $∥\text{Δ}A^{\text{comp}}{∥}_{1}$ is the sparsity penalty on the learnable compensation-aware topology, and *λ*_po_, *λ*_cf_, and *λ_s_*are weighting coefficients.

In the final configuration, $L_{\text{po}}$ and $L_{\text{cf}}$ were Smooth L1 losses. The sparsity constraint limited the density of the compensation-aware residual graph, thereby maintaining an inspectable topology. The objective jointly optimised both rehabilitation scores while regularizing the learned compensation topology.

### 3.7 Training strategy and baseline methods

All experiments were implemented in PyTorch with Python 3.13.11 and conducted on a workstation with one NVIDIA GeForce RTX 5090 GPU. We trained CTCG-Net for 100 epochs using AdamW, an initial learning rate of 5×10^−4^, a weight decay of 1×10^−4^, and a mini-batch size of 16. The learning rate decreased stepwise at epochs 30, 50, and 70. The backbone used three-channel skeletal inputs, a dropout rate of 0.35, mixed-precision training, gradient clipping at a norm of 5.0, and edge-importance weighting.

Training followed the multitask objective in Equation 7. Both regression branches used Smooth L1 loss, with sparsity regularisation applied to the compensation-aware graph residual. The final configuration used *λ*_po_ = 1.0, *λ*_cf_ = 1.0, and *λ_s_*= 1 × 10^−4^. The Results section reports a sensitivity analysis of alternative weights. Equal PO and CF weights preserved balanced optimisation of the predefined targets, whereas Smooth L1 loss reduced the influence of outlying errors. All experiments used identical temporal alignment and 512-frame resampling procedures. Performance differences could therefore be attributed primarily to model architecture rather than input preparation.

The baselines covered conventional graph convolution, advanced spatio-temporal graph variants, transformer-based movement modelling, and recurrent sequence learning. We compared CTCG-Net with CMR, CMT, ST-GCN, CTR-GCN, MS-G3D, PoseC3D, Shift-GCN, TST, and BiLSTM (Liao et al., 2020a; Yao et al., 2023; Deb et al., 2022; Mourchid and Slama, 2023; Yu et al., 2024; Yan et al., 2018; Chen et al., 2021; Liu et al., 2020; Duan et al., 2022; Cheng et al., 2020; Zhang et al., 2020; Song et al., 2021). We adapted every baseline to the same dual-output PO and CF regression setting. Each backbone used the Spatially Decoupled Regression Head of CTCG-Net, which isolated differences in spatio-temporal feature extraction and multimodal representation from differences in the scoring head. All methods used identical Kinect skeletal and IMU inputs, preprocessing, temporal alignment, 512-frame resampling, participant-level data splits, and evaluation metrics. Hyperparameters were selected exclusively on the validation set, and the held-out test set was reserved for final reporting. We also recorded model capacity and computational cost when applicable.

### 3.8 Validation strategy and evaluation metrics

To prevent information leakage, we partitioned all data at the participant level. After integrity screening, the analytic cohort comprised 126 participants. The training, validation, and test sets contained 101, 13, and 12 participants, respectively, approximating an 8:1:1 ratio. All trials from each participant remained in one partition. Model development and hyperparameter selection used the training and validation sets, whereas final reporting used only the held-out test set.

We evaluated model performance using mean absolute deviation (MAD), root mean square error (RMSE), mean absolute percentage error (MAPE), and the Pearson correlation coefficient (*r*). Lower MAD, RMSE, and MAPE values indicate smaller prediction errors, whereas a higher *r* indicates stronger linear correspondence between predicted and reference scores.

For each target, *y_j_* denotes the reference score for test trial *j*, and ${\hat{y}}_{j}$ denotes the corresponding prediction. The number of test trials is n, and $\bar{y}$ and $\bar{\hat{y}}$ are the mean reference and predicted scores, respectively. We calculated the four metrics as follows:

(8) $$\begin{array}{l} \text{ MAD = }\frac{1}{n}\sum_{j=1}^{n}|{\hat{y}}_{j}-y_{j}|,\\ \text{ RMSE = }\sqrt{\frac{1}{n}\sum_{j=1}^{n}{\left({\hat{y}}_{j}-y_{j}\right)}^{2}},\\ \text{MAPE = }\frac{100}{n}\sum_{j=1}^{n}\left|\frac{{\hat{y}}_{j}-y_{j}}{y_{j}}\right|,\\ \;r\;=\;\frac{\sum_{j=1}^{n}\left({\hat{y}}_{j}-\bar{\hat{y}})(y_{j}-\bar{y}\right)}{\sqrt{\sum_{j=1}^{n}{\left({\hat{y}}_{j}-\bar{\hat{y}}\right)}^{2}}\sqrt{\sum_{j=1}^{n}{\left(y_{j}-\bar{y}\right)}^{2}}}. \end{array}$$

We applied these definitions consistently to PO and CF to compare error magnitude and linear correspondence within the same held-out participant-level test partition.

Because global correlation alone is insufficient for quantitative rehabilitation assessment, we also examined agreement on the held-out test set. Predicted-versus-reference scatter plots and Bland–Altman plots were generated for PO and CF. These analyses complemented error-based metrics by showing overall correspondence, systematic bias, and trial-level error dispersion across the score range.

### 3.9 Statistical analysis

Descriptive statistics summarised demographic characteristics and valid-trial distributions across exercise types. Continuous variables are reported as the mean ± standard deviation, and categorical variables as counts and percentages.

MAD, RMSE, MAPE, and the Pearson correlation coefficient (*r*) were calculated on the held-out test set using Equation 8. Agreement was analysed separately for PO and CF using predicted-versus-reference scatter plots and Bland–Altman plots. Mean bias and 95% limits of agreement were calculated as the mean paired difference ± 1.96 standard deviations of the paired differences.

Exercise-specific analyses descriptively assessed performance across the five rehabilitation tasks. The public dataset lacks complete stratification variables, including clinical severity, affected side, rehabilitation stage, and inter-rater reliability. We therefore did not conduct subgroup analyses based on these factors. All statistical summaries and visualisations used the same fixed training, validation, and test partition.

## 4 Results

### 4.1 Cohort and data characteristics

The final analysis used the public multimodal benchmark described in Section 3.2. The repository contains records from 128 participants, but two were excluded because incomplete multimodal files prevented synchronised input construction (Islam, 2024). Screening and preprocessing retained 126 participants and 558 valid exercise trials. We divided the dataset into training, validation, and test sets at the participant level using an 8:1:1 ratio. All trials from each participant remained in one partition to prevent participant-level information leakage.

**Table 2** summarises the analytic cohort and valid multimodal trials. The training, validation, and test sets contained 101, 13, and 12 participants and 440, 63, and 55 trials, respectively. Trial counts were broadly balanced across exercises, although Exercise Type 4 had the fewest valid trials. The benchmark includes upper-limb-dominant, trunk-oriented, pelvic-control, and lower-limb functional movements. It therefore provides heterogeneous movement patterns for evaluating multimodal rehabilitation assessment. Further stratification by stroke severity, lesion side, or rehabilitation stage was not possible because the release provides only participant identifier, age, and sex.

**Table 2:** Cohort characteristics, participant-level partition, and exercise-specific trial distribution in the final analytic dataset.

Characteristic	Overall	Train	Validation	Test
Participants, n	126	101	13	12
Age, years	22.4 ± 3.2	22.6 ± 2.8	20.6 ± 5.9	22.2 ± 1.7
Female, n (%)	54 (42.9)	45 (44.6)	4 (30.8)	5 (41.7)
Male, n (%)	72 (57.1)	56 (55.4)	9 (69.2)	7 (58.3)
Valid multimodal trials, n	558	440	63	55
Exercise type 1 trials, n	120	96	13	11
Exercise type 2 trials, n	117	93	13	11
Exercise type 3 trials, n	113	90	13	10
Exercise type 4 trials, n	99	75	13	11
Exercise type 5 trials, n	109	86	11	12

**Figure 3:**
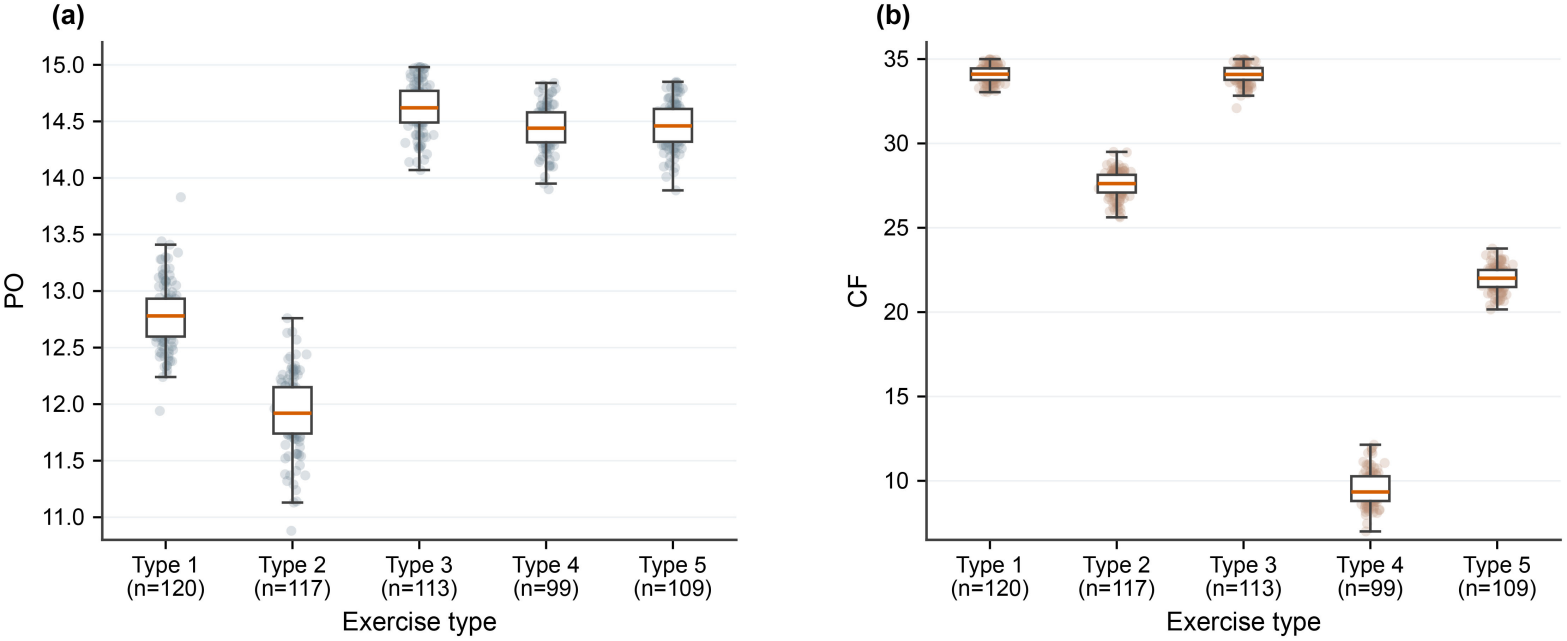
Reference-score distributions across the five rehabilitation exercises in the final analytic dataset. **(a)** Primary Outcome (PO). **(b)** Control Factor (CF). Each point represents a valid multimodal trial; box plots show the median and interquartile range.

**Figure 3** shows the reference-score distributions across the five exercise types. Both PO and CF varied across exercises. PO scores were generally lower and more dispersed for Types 1 and 2, whereas Types 3–5 had higher central tendencies and narrower distributions. CF followed a different pattern: values were higher for Types 1 and 3, intermediate for Type 2, lower for Type 5, and lowest for Type 4. The benchmark therefore comprises movements with different score ranges, compensation burdens, and label dispersion rather than a uniform regression task.

### 4.2 Main performance comparison

CTCG-Net achieved the lowest average errors among the evaluated methods in the target-aggregated comparison (**Table 3**). The average MAD, RMSE, and MAPE were 0.4001, 0.5026, and 2.2784%, respectively. These errors were lower than those of the standard ST-GCN. Because every baseline used the same PO and CF regression head and preprocessing pipeline, the comparison primarily reflects differences in the learned movement representations.

**Table 3:** Algorithm performance under the unified PO and CF assessment setting.

Metric	Exercise	Ours	ST-GCN	CMR	CMT	CTR-GCN	MS-G3D	PoseC3D	Shift-GCN	TST	BiLSTM
MAD (↓)	Ex1 Ex2 Ex3 Ex4 Ex5	0.2895 0.6206 0.3164 0.3891 0.3845	1.1422 1.0324 1.3111 1.8923 0.8764	0.9214 1.4520 1.0583 1.4812 0.8518	1.1248 1.1561 1.4123 1.8105 0.9175	1.6512 1.4381 1.8529 2.4173 1.5505	1.9573 2.6518 1.7482 2.8716 1.4421	2.6012 2.2450 2.5471 3.4508 1.9369	3.1092 3.4561 2.9515 3.1078 2.7505	4.1027 3.2184 3.6520 4.5162 2.8702	4.4315 2.7163 6.4109 5.6142 4.0371
	Average	0.4001	1.2509	1.1529	1.2842	1.7820	2.1342	2.5562	3.0750	3.6719	4.6420
RMSE (↓)	Ex1 Ex2 Ex3 Ex4 Ex5	0.3548 0.7677 0.4324 0.4853 0.4726	1.3894 2.0411 1.6075 3.8246 1.5353	1.0281 1.8516 1.4583 1.9512 1.1274	1.3572 1.7148 1.8512 2.1487 1.1631	1.9582 2.3041 2.5687 2.8915 1.7635	2.3456 2.9572 2.1093 3.8052 1.7681	3.1083 2.5489 3.4562 4.6517 4.6517	3.8514 3.6510 3.2546 4.2153 3.0374	4.5081 3.8592 4.2185 5.4120 3.2019	5.1032 2.8351 6.6520 6.2045 4.7182
	Average	0.5026	2.0796	1.4833	1.6470	2.2972	2.5971	3.1883	3.6019	4.2399	5.1026
MAPE (%) (↓)	Ex1 Ex2 Ex3 Ex4 Ex5	1.3401 3.0590 1.2161 3.8280 1.9488	2.4584 2.6249 2.7172 7.7590 2.3874	2.1581 2.8519 2.6582 5.1024 2.8516	2.8514 3.1082 3.5461 6.2153 2.5681	4.1082 3.8516 4.6518 9.2154 3.7915	5.8513 6.1085 4.2172 11.2581 4.3418	8.1025 5.8591 7.6583 12.5480 5.1124	10.2174 9.5482 8.4561 11.2152 7.8415	12.5481 8.8516 11.2145 14.6512 9.0182	9.4752 6.8614 13.1531 23.4918 11.0527
	Average	2.2784	3.5894	3.1244	3.6578	5.1237	6.3554	7.8561	9.4557	11.2567	12.8068

All baseline backbones used the same Spatially Decoupled Regression Head, input modalities, preprocessing, participant-level partition, and evaluation metrics.

The advantage of CTCG-Net was most apparent when accuracy and consistency were considered jointly. Several baselines performed competitively on individual exercises but varied more across tasks. CTCGNet maintained lower average errors across upper-limb, trunk-oriented, pelvic-control, and lower-limb movements. **Table 3** aggregates both targets to compare the overall scoring capability of each algorithm under the same multimodal benchmark conditions.

### 4.3 Validation stability across random seeds and subject-level folds

**Figure 4:**
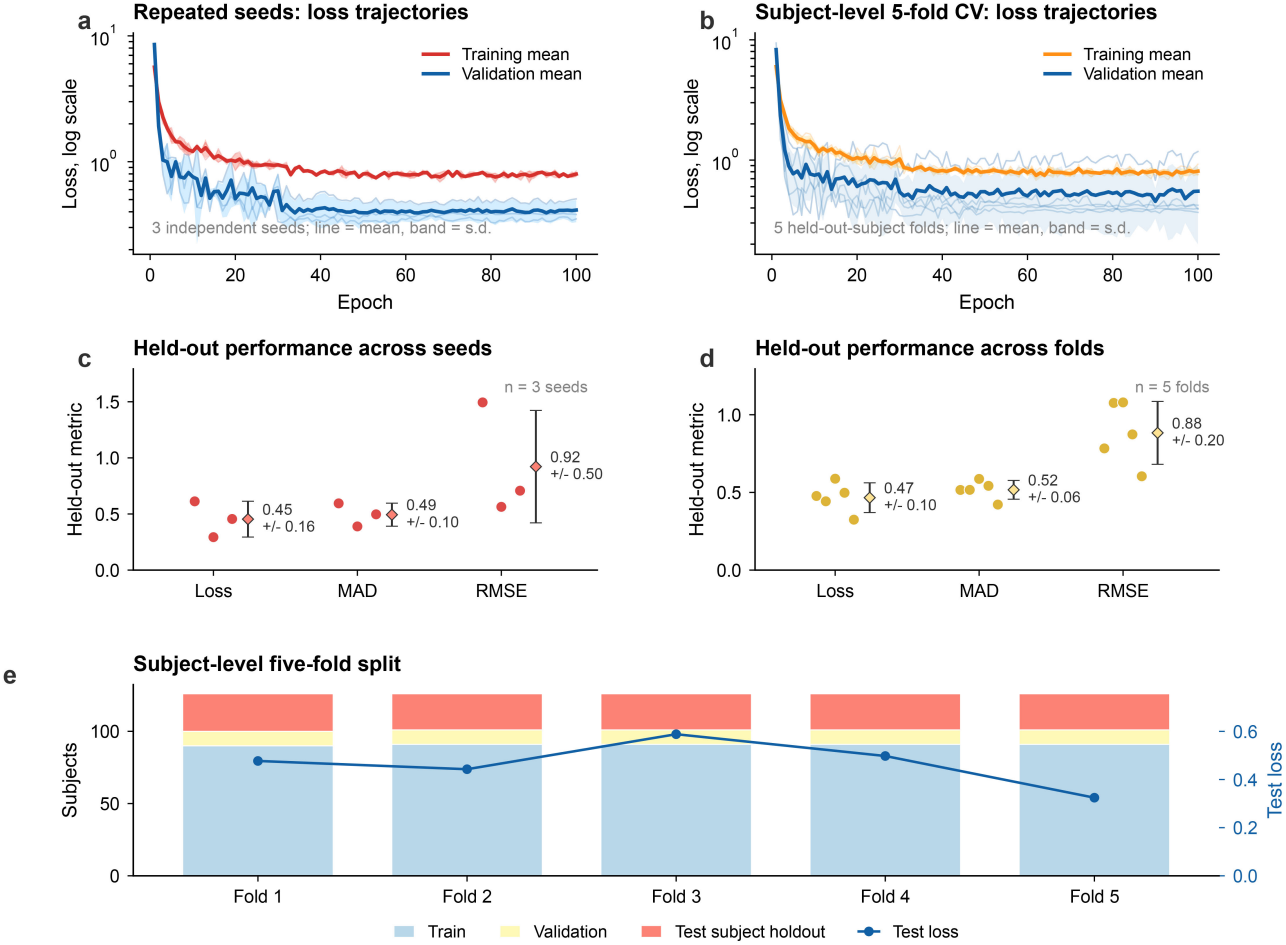
Training convergence and validation stability of CTCG-Net. **(a)** Training and validation loss across three random seeds. Thin lines show individual runs, bold lines show the mean, and shaded bands show the standard deviation. **(b)** Loss trajectories under participant-level five-fold cross-validation. **(c)** Held-out loss, MAD, and RMSE across random seeds. **(d)** Held-out metrics across participant-level folds. **(e)** Participant counts in each partition and fold-level held-out loss.

Because the main comparison used one held-out participant-level partition, we assessed sensitivity to stochastic training and alternative participant partitions. Training and validation losses decreased during early epochs and then plateaued without divergence (**Figure 4**). Across three random seeds, the held-out loss, MAD, and RMSE were 0.454 ± 0.160, 0.494 ± 0.103, and 0.922 ± 0.501, respectively. Participant-level five-fold cross-validation yielded corresponding averages of 0.466 ± 0.096, 0.517 ± 0.060, and 0.883 ± 0.202. These analyses were consistent with the main performance trend. However, the relatively wide RMSE variation indicates residual uncertainty in this small, heterogeneous benchmark.

### 4.4 Exercise-wise performance

**Figure 5:**
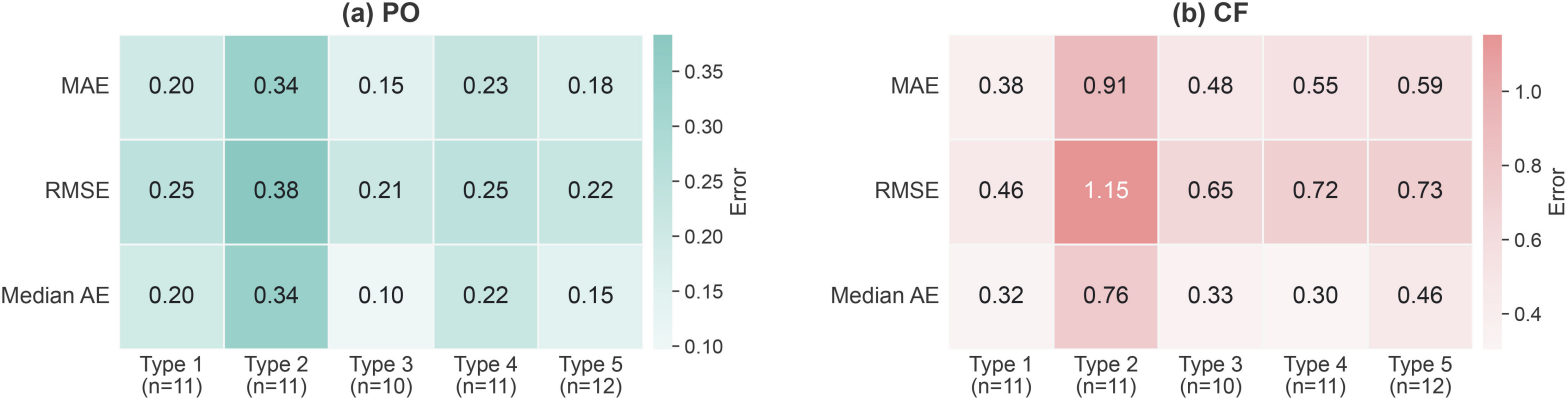
Exercise-specific prediction errors for the five rehabilitation tasks in the test set. **(a)** PO errors. **(b)** CF errors. Each panel reports MAE, RMSE, and median absolute error; darker shading indicates larger errors.

The exercise-specific heat maps showed different error profiles for PO and CF (**Figure 5**). Type 2 had the largest errors in the held-out test set, particularly for CF, whereas Type 3 had the smallest PO errors. Compensation-related scoring therefore varied in difficulty across exercises. This variation may reflect participant heterogeneity, task-specific postural demands, and limited observation of some compensatory patterns by the available sensors.

### 4.5 Agreement analysis and error dispersion

**Figure 6:**
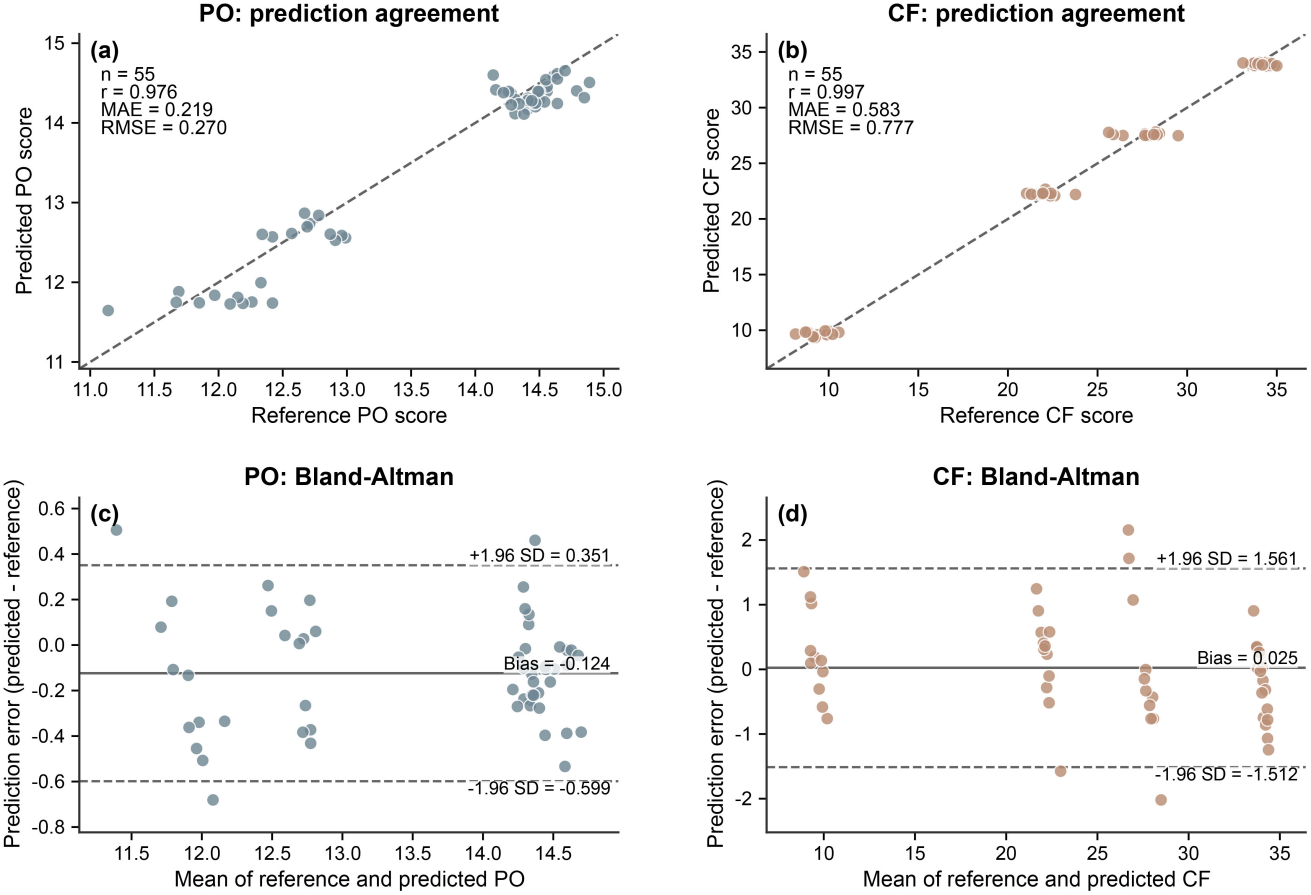
Prediction and agreement analyses for the held-out test set. **(a, b)** Predicted-versus-reference scores for PO and CF. **(c, d)** Bland–Altman plots showing mean bias and 95% limits of agreement for PO and CF.

Agreement analysis assessed whether overall predictive accuracy was accompanied by trial-level consistency. Predicted-versus-reference scatter plots show the correspondence between outputs and reference scores (**Figure 6**). Bland–Altman plots characterise systematic bias and error dispersion across the score range. This distinction is important because high correlation does not necessarily indicate close agreement for individual trials.

PO predictions corresponded closely with the reference scores and showed relatively narrow Bland– Altman limits. CF predictions also had high correlation, but trial-level errors were more dispersed. This finding is consistent with the greater spatial and postural complexity of compensation-related scoring. PO achieved *r* = 0.976, MAE = 0.219, and RMSE = 0.270. CF achieved *r* = 0.997, MAE = 0.583, and RMSE = 0.777.

### 4.6 Ablation study

We used an ablation study to assess the contributions of the Prior-Guided ST-GCN and Kinematic Relay Gating while retaining the Spatially Decoupled Regression Head. The decoupled-head baseline without either module produced larger prediction errors (**Table 4**). Addition of either module reduced error, and the complete configuration achieved the lowest errors (MAD = 0.4001; RMSE = 0.5026; MAPE = 2.2784%; *r* = 0.9978). These results indicate complementary contributions of the compensation-aware topology and relay gating within the decoupled-head configuration.

**Table 4:** Ablation results for the proposed modules.

Prior-guided ST-GCN	Kinematic relay gating	Spatially decoupled regression head	MAD (↓)	RMSE (↓)	MAPE (%) (↓)	Pearson *r* (↑)
– ✓ – ✓	– – ✓ ✓	✓ ✓ ✓ ✓	0.5259 0.4796 0.4136 0.4001	0.7700 0.6789 0.5917 0.5026	3.0405 2.7596 2.3275 2.2784	0.9962 0.9974 0.9977 0.9978

### 4.7 Loss-weight sensitivity analysis

We varied the PO, CF, and compensation-topology sparsity weights while holding the data partition, architecture, optimizer, and training schedule constant. **Table 5** reports one-factor sensitivity analyses around the final configuration. The selected values, *λ*_po_ = 1.0, *λ*_cf_ = 1.0, and *λ_s_*= 1 × 10^−4^, yielded the lowest MAD, RMSE, and MAPE among the evaluated settings. Increasing or decreasing either target weight increased the pooled error. Removing the sparsity penalty or increasing it to 1×10^−3^ also increased error. A small sparsity penalty and equal PO and CF weights were therefore appropriate for this held-out partition.

**Table 5:** Sensitivity of CTCG-Net to PO, CF, and sparsity weights.

Sensitivity axis	$\lambda_{\text{po}}$	$\lambda_{\text{cf}}$	$\lambda_{s}$	MAD ( ↓)	RMSE ( ↓)	MAPE (%) ( ↓)	Pearson r ( ↑)
PO weight	0.5	1.0	$1\times 10^{-4}$	0.4720	0.6389	2.5641	0.9973
PO weight, selected	**1.0**	**1.0**	$1\times 10^{-4}$	**0.4001**	**0.5026**	**2.2784**	**0.9978**
PO weight	2.0	1.0	$1\times 10^{-4}$	0.4648	0.6283	2.7051	0.9974
CF weight	1.0	0.5	$1\times 10^{-4}$	0.4234	0.5999	2.4538	0.9977
CF weight, selected	**1.0**	**1.0**	$1\times 10^{-4}$	**0.4001**	**0.5026**	**2.2784**	**0.9978**
CF weight	1.0	2.0	$1\times 10^{-4}$	0.4593	0.6419	2.6384	0.9973
Sparse weight	1.0	1.0	0	0.4923	0.6536	2.9265	0.9972
Sparse weight, selected	**1.0**	**1.0**	$1\times 10^{-4}$	**0.4001**	**0.5026**	**2.2784**	**0.9978**
Sparse weight	1.0	1.0	$1\times 10^{-3}$	0.4847	0.6597	2.7512	0.9973

Metrics were calculated on the held-out participant-level test set using pooled PO and CF predictions. Bold values denote the selected configuration in each one-factor analysis.Bold values indicate the best performance among the compared configurations.

### 4.8 Robustness under corrupted multimodal inputs

We assessed robustness under three test-time perturbations: random missing skeletal joints, additive skeletal-tracking noise, and IMU-channel dropout. These perturbations approximate practical sensing failures, including temporarily unobserved joints, noisy skeletal coordinates, and unavailable wearable channels. We did not repeat training; the same checkpoint was evaluated under each corrupted-input condition. **Table 6** uses the unified PO and CF protocol applied to the complete model in **Table 4**.

**Table 6:** Robustness of CTCG-Net to corrupted multimodal inputs.

Scenario	Test-time perturbation	MAD (↓)	RMSE (↓)	MAPE (%) (↓)	Pearson *r* (↑)
Clean	None	**0.4001**	**0.5026**	**2.2784**	**0.9978**
Missing joints	10% random joint masking	0.7361	1.4980	4.6175	0.9856
Skeleton noise	Gaussian noise, SD = 0.05	0.4004	0.5830	2.2889	0.9978
IMU dropout	20% random IMU-channel masking	0.4069	0.5886	2.3310	0.9977

Bold values denote the baseline performance under unperturbed multimodal input conditions, and indicate the best performance among all tested robustness scenarios.

The clean condition reproduced the complete-model results (MAD = 0.4001; RMSE = 0.5026; MAPE = 2.2784%; *r* = 0.9978). Skeletal noise and IMU dropout modestly increased RMSE to 0.5830 and 0.5886, respectively. By contrast, 10% random joint masking increased MAD to 0.7361, RMSE to 1.4980, and MAPE to 4.6175%, while *r* decreased to 0.9856. Missing skeletal joints were the most disruptive tested perturbation and may pose a practical deployment risk.

**Figure 7:**
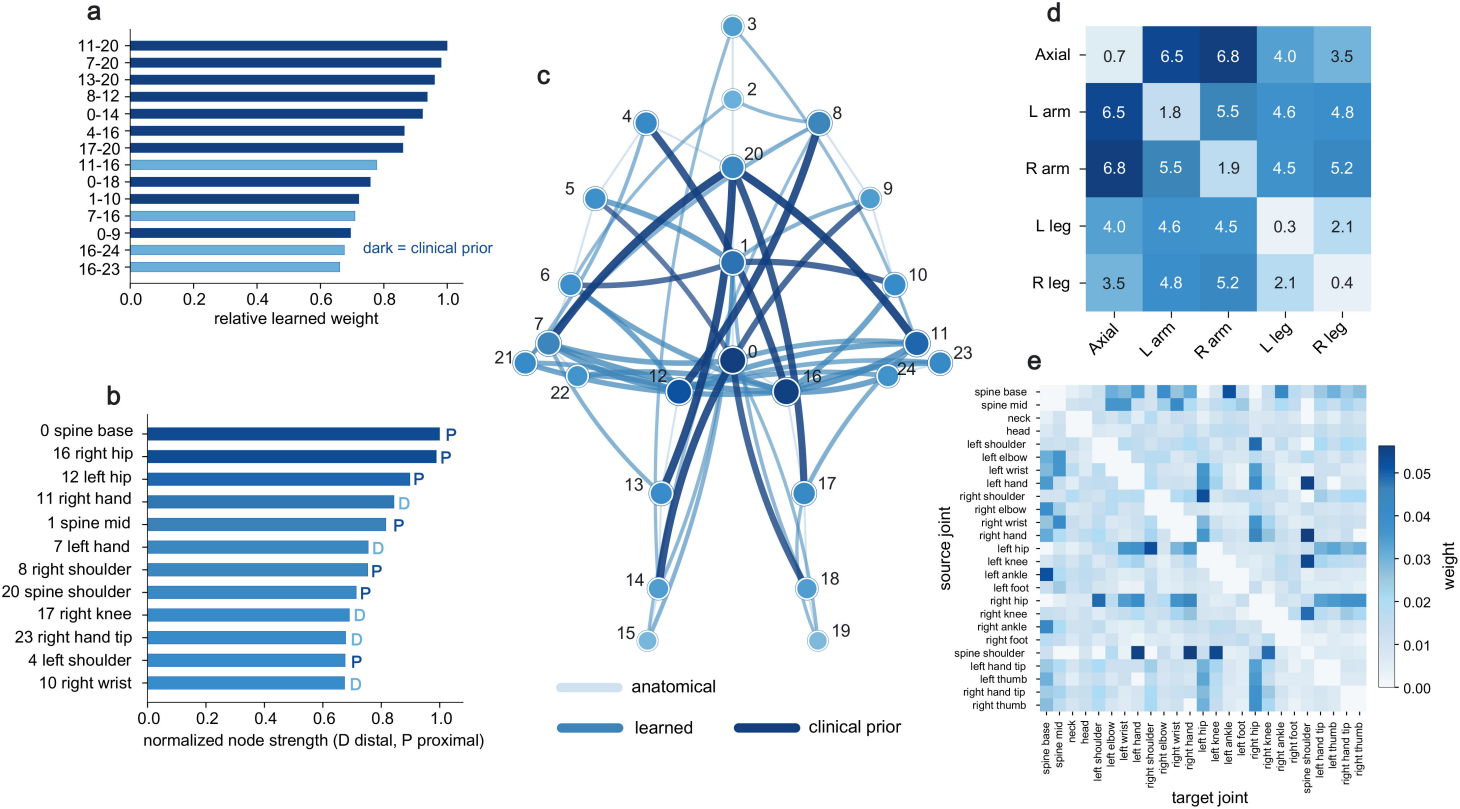
Training-derived compensation topology of CTCG-Net. **(a)** Highest-weight joint-to-joint edges; darker bars denote overlap with the clinical prior. **(b)** Normalised node strength. **(c)** Topology projected onto the skeleton. Light, medium-blue, and dark-blue lines denote anatomical, learned compensation-related, and prior-aligned learned edges, respectively. **(d)** Topology mass across body modules. **(e)** Complete joint-by-joint topology matrix. The topology emphasises distributed cross-regional coordination rather than only local anatomical connections.

We visualised the training-derived topology from several perspectives to examine the compensation-aware component (**Figure 7**). This analysis identified the dominant joint associations and the body regions with the greatest topology mass. It also assessed whether the learned structure was compatible with clinically plausible compensation patterns.

The learned graph showed stronger long-range associations involving the axial skeleton, shoulder girdle, pelvis, and proximal-to-distal connections. Some learned edges overlapped with the clinical prior. These patterns are consistent with the intended representation of distributed coordination by the residual topology.

### 4.9 Interpretability analysis

We generated branch-specific saliency maps for representative trials to examine the internal responses of the two-dimensional design. The analysis assessed whether the PO and CF branches attended to different spatial regions during the same movement. These qualitative results complement the numerical evaluations in **Tables 3**, **4**.

The saliency patterns represent model attributions rather than biomechanical evidence or proof of clinical interpretability. They can indicate whether the branches attend to different regions but cannot replace quantitative attribution-consistency analysis, clinician feedback, or prospective evaluation. We therefore used them only to assess compatibility between the learned representations and therapist-oriented reasoning. A PO-dominant response was expected near effector and task-execution regions, whereas a CF-dominant response could involve broader trunk, shoulder-girdle, pelvic, and postural-control regions.

**Figure 8:**
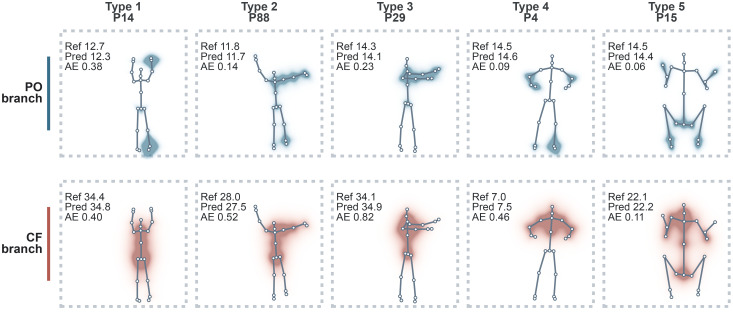
Representative saliency maps for the PO and CF branches across five exercises. One test trial per exercise was used for paired branch visualisation. Joint-level gradient saliency was overlaid on the skeleton, with greater intensity indicating a larger contribution to the branch output. Each panel shows the reference score, prediction, and absolute error.

The PO branch generally produced localised responses around task-execution regions (**Figure 8**). The CF branch showed broader saliency across the trunk, shoulder girdle, pelvis, or whole body, depending on the exercise. These qualitative patterns indicate that the branches used different spatial evidence but do not establish causal movement mechanisms.

## 5 Discussion

We developed CTCG-Net, a multimodal spatio-temporal graph framework for therapist-assisted stroke rehabilitation assessment (Lim et al., 2026; Rahman et al., 2023; Khalaf et al., 2026; Bai et al., 2024; Geed et al., 2023). Compensation-aware graph modelling reduced prediction errors relative to the evaluated graph, sequence, and action-quality baselines. Inertial-guided relay gating incorporated local dynamic information while preserving the skeletal structure. Spatially decoupled regression enabled separate estimation of the related PO and CF targets. Sensitivity and corrupted-input analyses further characterised configuration stability and identified missing skeletal joints as the principal tested deployment risk.

The framework reflects the clinical distinction between task completion and movement strategy (Santisteban et al., 2016; Liao et al., 2020b; Wu et al., 2025). A successfully completed movement may still depend on trunk substitution, proximal stabilisation, or abnormal postural adjustment. Separating PO from CF aligns the learning targets more closely with therapist assessment. It may also prevent high task completion from being interpreted as necessarily indicating high-quality movement control. PO showed closer trial-level agreement than CF, which had greater error dispersion. This difference is consistent with the greater spatial and postural complexity of compensation-related scoring.

The model components address complementary aspects of the assessment problem. The compensation-aware residual topology extends anatomical graph convolution to represent cross-regional coordination. The learned topology emphasised axial, shoulder-girdle, pelvic, and distal-to-proximal relationships. Relay gating uses IMU dynamics to regulate skeletal features while retaining the skeletal graph as the primary structured representation. Spatially decoupled regression then directs PO and CF estimation towards different spatial evidence. The ablation results indicate that these components jointly contributed to the performance of the complete model.

This study has several limitations (Zhu et al., 2019; Sardari et al., 2024; Ramírez-Sanz et al., 2023; Li et al., 2025; Pohl et al., 2022). First, the public dataset lacks detailed information on severity, lesion side, rehabilitation stage, and inter-rater reliability. The benchmark also provides insufficient information to verify the clinical validity of the PO and CF labels. Missing details include rater background, scoring procedures, blinding, disagreement resolution, and inter-rater reliability. The scores should therefore be considered benchmark supervisory labels rather than fully validated clinical ground truth. Second, two IMUs reduce wearable burden but cannot capture local inertial dynamics across all body regions. Third, fixed-length resampling supports offline benchmarking but not variable-length or streaming assessment. Fourth, robustness testing used simulated perturbations rather than prospectively recorded sensor failures. Finally, the model lacked external validation across institutions, devices, and patient populations.

Future studies should evaluate CTCG-Net in prospective cohorts with richer clinical metadata and repeated therapist ratings. External-device and external-site validation and methods for missing-joint and variable-length inference are also needed. Quantitative attribution-consistency analysis and clinician feedback should establish whether the saliency outputs are useful in practice. Until this evidence is available, CTCG-Net should be considered a quantitative support model for clinician-supervised assessment and longitudinal monitoring, not an autonomous clinical decision system.

## 6 Conclusions

CTCG-Net is a multimodal spatio-temporal graph framework for therapist-assisted stroke rehabilitation assessment. It integrates a Prior-Guided ST-GCN, Kinematic Relay Gating, and a Spatially Decoupled Regression Head to estimate PO and CF separately from synchronised skeletal and inertial measurements.

On the public multimodal benchmark, CTCG-Net achieved the lowest average errors among the evaluated methods. Ablation analysis indicated contributions from the compensation-aware topology and relay-gating modules. Robustness testing identified missing skeletal joints as the principal tested perturbation risk. Agreement, topology, and saliency analyses provided bounded evidence of score correspondence and branch-specific spatial responses. CTCG-Net may provide reference scores for clinician-supervised review, but it remains an auxiliary research tool. Clinical validation, quantitative interpretability assessment, clinician-feedback studies, and external testing are required before broader rehabilitation use.

## Data Availability

The dataset analysed in this study is available from Mendeley Data at https://data.mendeley.com/datasets/ygpdzx52g2/1 (DOI: 10.17632/YGPDZX52G2.1).

## Glossary

BiLSTM: Bidirectional Long Short-Term Memory
CF: Control Factor
CTCG-Net: Compensatory-Topology and Relay-Gating Graph Network
EAAQ: Exercise Accuracy Assessment Questionnaire
IMU: Inertial Measurement Unit
MAD: Mean Absolute Deviation
MAPE: Mean Absolute Percentage Error
PO: Primary Outcome
RMSE: Root Mean Square Error
ST-GCN: Spatio-Temporal Graph Convolutional Network
